# Common variants of *ARID1A* and *KAT2B* are associated with obesity in Indian adolescents

**DOI:** 10.1038/s41598-018-22231-x

**Published:** 2018-03-02

**Authors:** Anil K. Giri, Vaisak Parekatt, Om Prakash Dwivedi, Priyanka Banerjee, Khushdeep Bandesh, Gauri Prasad, Nikhil Tandon, Dwaipayan Bharadwaj

**Affiliations:** 1 Genomics and Molecular Medicine Unit, CSIR-Institute of Genomics and Integrative Biology, Sukhdev Vihar, Mathura Road, New Delhi, 110025 India; 2 Academy of Scientific and Innovative Research, CSIR-Institute of Genomics and Integrative Biology Campus, Sukhdev Vihar, Mathura Road, New Delhi, 110025 India; 3 Department of Endocrinology and Metabolism, All India Institute of Medical Sciences, Ansari Nagar, New Delhi, 110608 India; 4 Present Address: Systems Genomics Laboratory, School of Biotechnology, Jawaharlal Nehru University, New Mehrauli Road, Munirka, Delhi, 110067 India

## Abstract

Obesity involves alterations in transcriptional programs that can change in response to genetic and environmental signals through chromatin modifications. Since chromatin modifications involve different biochemical, neurological and molecular signaling pathways related to energy homeostasis, we hypothesize that genetic variations in chromatin modifier genes can predispose to obesity. Here, we assessed the associations between 179 variants in 35 chromatin modifier genes and overweight/obesity in 1283 adolescents (830 normal weight and 453 overweight/obese). This was followed up by the replication analysis of associated signals (18 variants in 8 genes) in 2247 adolescents (1709 normal weight and 538 overweight/obese). Our study revealed significant associations of two variants rs6598860 (OR = 1.27, *P* = 1.58 × 10^–4^) and rs4589135 (OR = 1.22, *P* = 3.72 × 10^–4^) in *ARID1A* with overweight/obesity. We also identified association of rs3804562 (β = 0.11*, P* = 1.35 × 10^–4)^ in *KAT2B* gene with BMI. In conclusion, our study suggests a potential role of *ARID1A* and *KAT2B* genes in the development of obesity in adolescents and provides leads for further investigations.

## Introduction

Obesity in childhood and adolescence can lead to life-long complications on individual’s health. Obese and overweight adolescents are more likely to have respiratory, sleep-related, behavioral and mental health problems^1^. Childhood obesity can also persist into adulthood, making such individuals vulnerable to developing diseases like cancer, arthritis, coronary heart disease and other chronic metabolic diseases such as type 2 diabetes^2,3^. There were 42 million overweight children (below 5 years) across the globe in 2015^4^. By contributing to the growing prevalence of associated complications, the childhood obesity is putting an increasing burden on the global public health systems^5^. Hence it is necessary to study the predisposing factors associated with it.

Certain populations have a stronger genetic predisposition to obesity compared to other populations^6^, with varying degree of susceptibility among individuals within a population^7^. The available literature suggests a strong genetic basis of obesity^8–11^. Genetic studies have implicated 227 genetic variants from genes involved in various signaling pathways (neuro-endocrine coordination, insulin signaling, lipid metabolism, adipocyte differentiation, muscle and liver biology, maintenance of gut microbiota) involved in the etiology of common polygenic obesity^9^. The environment also plays a significant role in the development of obesity^12^. Excessive food intake and insufficient physical activity can disrupt body’s energy homeostasis^13^. This disruption can lead to changes in complex biochemical and signaling pathways involved in the alimentary, neuroendocrine and immune system. Such changes in the internal environment of the body manifest at cellular level in the form of altered transcriptional programs. Recent research efforts have shown that chromatin modifications allow cells to make rapid and context-specific transcriptional changes^13^. Several proteins have been identified as components of chromatin modification complexes that can control the accessibility of DNA towards transcription factors, in effect controlling transcription in various disease states^14^. It is therefore important to understand the role of chromatin modification with respect to normal or disease states including obesity.

In the context of obesity, a few chromatin modifying proteins have been identified those play roles in controlling transcriptional rewiring in response to environment^15,16^. A United Kingdom-based study has detected mutations in *DNMT3A* (chromatin modifier gene) in children with overgrowth syndrome^17^. Functional studies to identify novel players are confounded by the fact that obesity is a systemic condition affecting multiple tissues. Different players may be involved in different tissues for the same purpose. This adds complexity to the identification of chromatin modifiers involved in obesity. Candidate gene-based association could provide simpler approaches to identify chromatin modifier genes involved in obesity. Here, we hypothesized that genetic variants in chromatin modifying genes could play an important role in the development of obesity. To test this hypothesis, we conducted the first candidate gene-based association study investigating chromatin modifying genes to identify genetic variants associated with adolescent obesity.

## Results

Anthropometric and clinical characteristics of study participants have been provided in Table 1. Association analysis in Stage 1 revealed the associations of 28 variants in 13 genes with overweight/obesity at *P* < 0.05 (Supplementary Table 1). The top signals for overweight/obesity were identified at rs4589135 [*P* = 1.2 × 10^−4^] and rs6598860 [*P* = 7.8 × 10^−4^] in *ARID1A* gene. Association analysis with BMI identified rs907092 in *IKZF3* (*P* = 3.6 × 10^−6^) as top signals in stage 1.

**Table 1:** Clinical status of study participants.

Character	Stage 1		Stage2		P*	*P* ^†^
	NW adolescents	OW/OB adolescents	NW adolescents	OW/OB adolescents		
N(male/female)	369/461	173/280	779/930	220/318		
Age (years)	14 (12.5–15.0)	13(12.0–15.0)	13(12.00–14)	13(12.0–15.0)	1.98 × 10^−28^	0.80
Height (m)	1.54(1.48–1.62)	1.56(1.50–1.62)	1.54(1.47–1.60)	1.57(1.51–1.63)	9.13 × 10^−3^	0.18
Z Height	−0.23 ± 2.42	0.54 ± 2.16	−0.08 ± 0.98	0.26 ± 0.97	0.09	0.04
Weight (kg)	43.08(37–48.73)	65.12(55.50–73.80)	42.70(36.0–49.0)	63(55.6–63.39)	0.88	0.08
Z Weight	−0.57 ± 0.86	1.18 ± 0.72	−0.40 ± 0.62	1.26 ± 0.79	5.2 × 10^−11^	0.24
BMI (kg/m^2^)	17.78(15.86–19.44)	26.52(23.97–28.93)	17.87(16.12–19.73)	25.27(23.68–27.44)	0.26	5.09 × 10^−4^
Z BMI	−0.60 ± 0.71	1.07 ± 0.59	−0.44 ± 0.53	1.35 ± 0.76	1.44 × 10^−12^	6.05 × 10^−6^
WC (m)	0.67(0.61–0.72)	0.84(0.77–0.90)	0.66(0.60–0.73)	0.84(0.76–0.77)	0.02	0.05
Z WC	−0.23 ± 0.54	0.81 ± 0.50	−0.73 ± 1.90	0.31 ± 0.29	8.71 × 10^−6^	4.65 × 10^−15^
HC (m)	0.81(0.76–0.86)	0.98(0.92–1.04)	0.80(0.74–0.86)	0.96(0.88–0.87)	0.09	2.8 × 10^−3^
Z HC	−0.13 ± 0.46	0.76 ± 0.42	−0.33 ± 0.80	0.30 ± 2.9	3.39 × 10^−15^	7.84 × 10^−21^
WHR	0.82(0.78–0.87)	0.86(0.81–0.90)	0.82(0.76–0.87)	−0.79(0.87–0.92)	2.38 × 10^−4^	5.73 × 10^−8^
Z WHR	0.16 ± 0.32	0.26 ± 0.27	−0.1.26 ± 3.05	−0.88 ± 0.2.9	4.79 × 10^−32^	1.61 × 10^−8^

Data are represented as median (inter-quartile range). Mann-Whitney U test was used to compare the medians. Calculated Z-scores are shown as mean z score + SD and were compared by student’s t test. *P values for comparison between NW adolescents from stage 1 and NW adolescents from stage 2. ^†^P values for comparison between OW/OB adolescents from stage 1 and OW/OB adolescents from stage 2.

**Figure 1:**
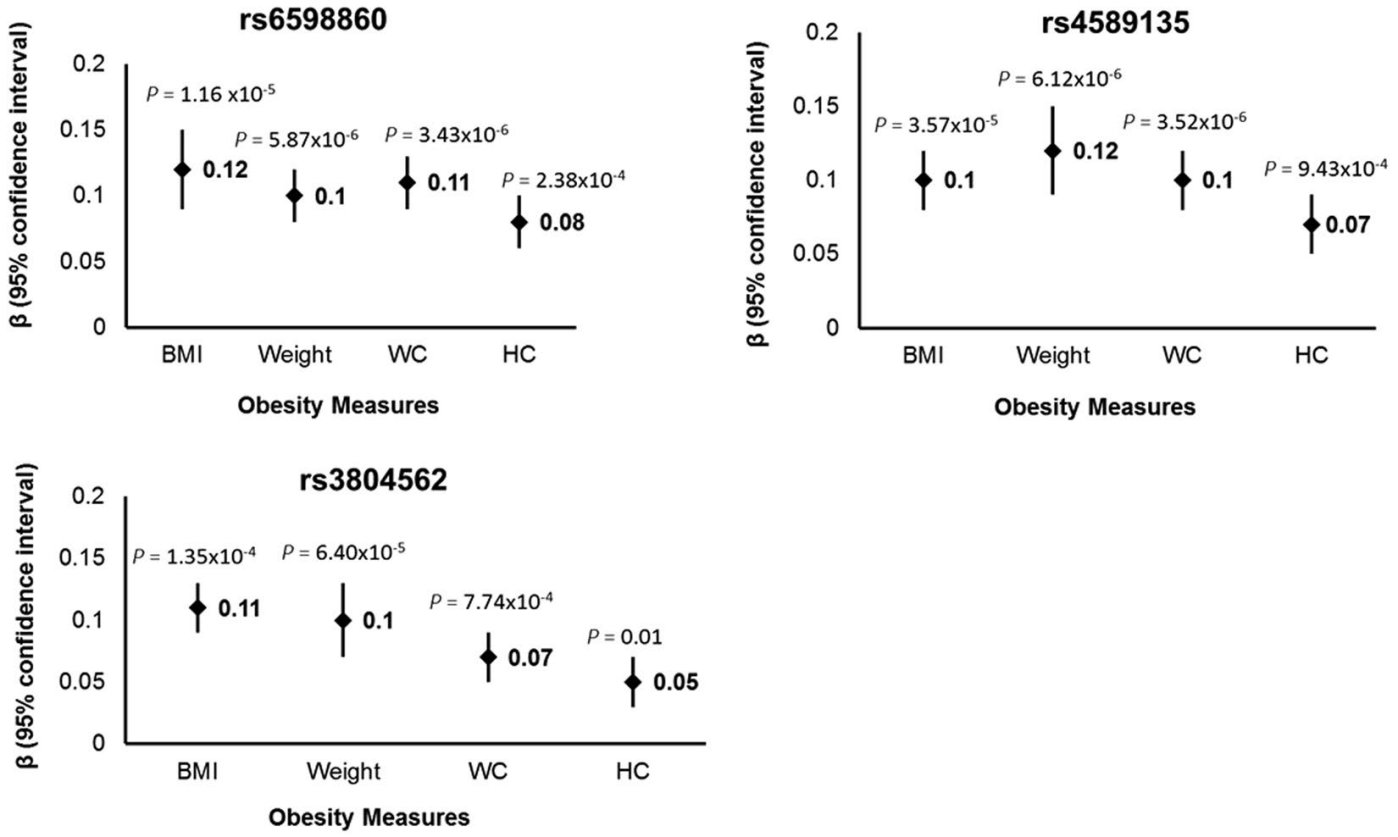
Associations of significant SNPs with measures of obesity. Association of overweight/obese associated SNPs with anthropometric measures of obesity (weight, BMI, WC, HC) in meta-analysis results. The z score change per risk allele for associated SNPs in meta-analysis has been plotted against corresponding phenotypes.

**Figure 2:**
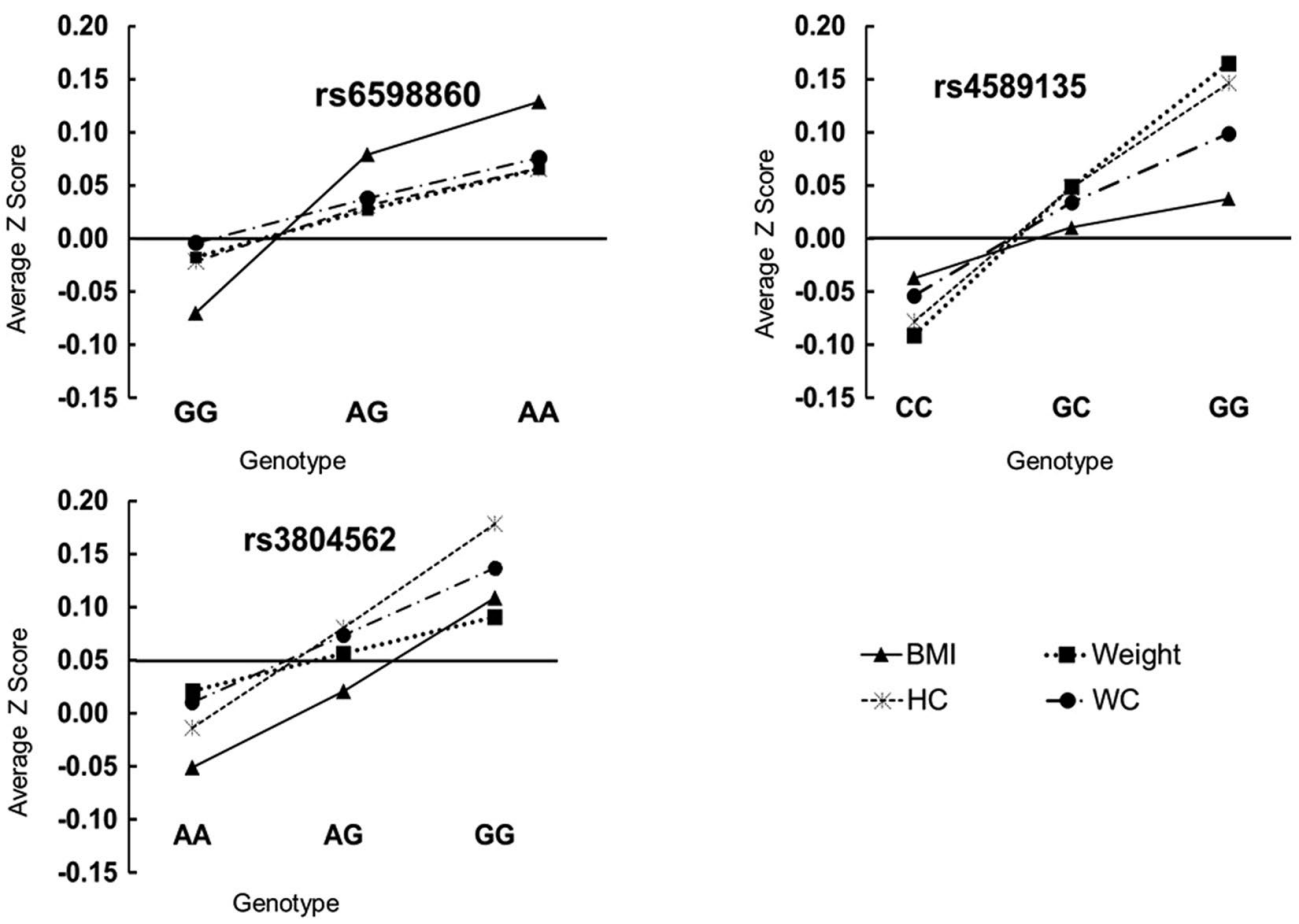
Effect of genotype of significant SNPs over z score of adiposity measures. Variation of adiposity measures with the different genotypes of associated SNPs. The average z score is plotted on the y-axis against the different genotypes of SNPs on the x-axis for SNPs associated with adiposity measures. The analysis has been performed on total samples obtained after combining samples from stage 1 and stage 2.

We successfully genotyped 18 SNPs in stage 2 and 13 SNPs passed the QC (Supplementary Table 2). We replicated the associations of rs6598860 (*P* = 0.04) in *ARID1A* and rs17003998 in *SMARCE1* (*P* = 0.01) with overweight/obesity (Table 2). Subsequent meta-analysis of summary results from stage 1 and stage 2 revealed significant association of rs6598860 (*P* = 1.58 × 10^−4^) and rs4589135 in *ARID1A* (*P* = 3.72 × 10^−4^) with overweight/obesity after multiple testing correction (*P* = 6.33 × 10^−4^). Meta-analysis of BMI data showed significant association of rs3804562 (*P = *1.35 × 10^−4^) in *KAT2B* along with rs4589135 (*P* = 3.57 × 10^−5^) and rs6598860 (*P* = 1.16 × 10^−5^) in *ARID1A* after multiple corrections (Table 2). Identified variants were also associated with other adiposity measures (Fig. 1, Supplementary Table 3). Variations in adiposity measures according to different genotypes of identified SNPs have been shown in Fig. 2. The study has more than 80% power to detect an association of variant with an observed allele frequency of 0.30 and an effect size of 1.20–1.30 (Supplementary Figure 1).

**Table 2:** Association of significant SNPs with obesity and BMI.

OBESITY											ZBMI					
STAGE1				STAGE 2			META-ANALYSIS				STAGE 1		STAGE 2		META-ANALYSIS	
SNP (RA/AA) Gene	RAF OW/OB, NW	OR (95%CI)	P	RAF OW/OB, NW	OR (95%CI)	P	OR	P	HetPVal	β(SE)	P	β(SE)	P	β(SE)	P	HetPVal
rs6598860(A/G); *ARID1A*	0.31,0.24	1.37 (1.36–1.37)	7.8 × 10^–4^	0.29, 0.25	1.19 (1.01–1.4)	0.04	1.27 (1.14–1.39)	1.58 × 10^–4^	0.26	0.17 (0.05)	2.2 × 10^−4^	0.09 (0.03)	0.01	0.12 (0.03)	1.16 × 10^–5^	0.18
rs4589135 (G/A); *ARID1A*	0.43, 0.35	1.41 (1.40–1.42)	1.2 × 10^–4^	0.41, 0.38	1.11 (0.96–1.28)	0.15	1.22 (1.08–1.41)	3.72 × 10^–4^	0.04	0.15 (0.04)	6.0 × 10^–4^	0.08 (0.03)	0.01	0.1 (0.02)	3.57 × 10^–5^	0.20
rs3804562 (C/T); *KAT2B*	0.57, 0.53	1.18 (1.17–1.18)	0.04	0.59, 0.55	1.14 (0.99–1.32)	0.08	1.15 (1.03–1.32)	8.93 × 10^–3^	0.76	0.11 (0.04)	7.37 × 10^−3^	0.11 (0.02)	6.20 × 10^–4^	0.11 (0.02)	1.35 × 10^–4^	0.86

RA = Risk Allele, AA = Alternate Allele, RAF = Risk allele frequency, OW/OW = Overweight/obese individuals, HetPVal = Heterogeneity p value for effect of the SNP in 1^st^ phase and 2^nd^ phase of individuals, Meta-analysis was performed using inverse variance model using fixed effect model using METAL. The OR and beta value presented here are with respect to risk alleles.

We searched for the associations of identified variants with obesity-related phenotypes in Genetic Investigation of Anthropometric Traits (GIANT)^18^ consortium data. The analysis revealed that all the identified SNPs are associated with either BMI or other related traits (Table 3) with similar effect sizes at *P* <  = 0.05.

**Table 3:** Association status of significant SNPs with obesity and other related parameters in GIANT consortium data.

Gene	SNP	Chr	Position_hg19	RA/OA	Sample_size	Effect	Standard error	P	Trait	Sample group
*ARID1A*	rs6598860	1	27041714	A/G	253288	0.01	0.005	0.04	BMI	Mixed ancestry individuals
*ARID1A*	rs4589135	1	27041714	C/T	75552	0.02	0.0056	5.03 × 10^–3^	WHR	Women of mixed ancestry adjusted for physical activity
*ARID1A*	rs4589135	1	27041714	C/T	71104	0.02	0.0058	0.01	WHR	European women adjusted for physical activity
*ARID1A*	rs4589135	1	27041714	C/T	58558	0.02	0.0063	0.02	WHR adjusted BMI	Physically active woman of mixed ancestry
*ARID1A*	rs4589135	1	27041714	C/T	71426	0.01	0.0057	0.03	BMI	Physically active man of mixed ancestry
*ARID1A*	rs4589135	1	27041714	C/T	55519	0.01	0.0065	0.05	WHR adjusted for BMI	Physically active European women
*ARID1A*	rs4589135	1	27041714	C/T	103871	0.01	0.0049	0.05	WC adjusted for BMI	Physically active European
*KAT2B*	rs3804562	3	20181028	T/C	52137	0.02	0.0091	0.03	WHR adjusted for BMI and physical activity	A ancestry men
*KAT2B*	rs3804562	3	20181028	T/C	126190	0.01	0.0063	0.05	WHR adjusted for BMI and physical activity	Mixed ancestry individuals

Chr: Chromosome, P: P value. RA: Risk allele, OA: Other allele. The summary statistics presented were obtained from the publicly available data from The Genetic Investigation of Anthropometric Traits (GIANT) consortium. The effect sizes are reported with respect to RA.

## Discussion

The study has used an already established cohort for children/adolescent obesity for its finding. There were 1280 and 863 common samples between current studies and our earlier work investigating the association of common variants in inflammatory marker genes with overweight or obesity in Indian children/adolescents by Tabassum *et al*.^19^ in stage 1 and 2 respectively. Our results demonstrate the associations of two SNPs in *ARID1A* (rs6598860 and rs4589135) with the risk of overweight/obesity in urban Indian adolescents. A moderate LD (R^2^ = 0.57) between rs6598860 and rs4589135 was observed in combined samples from stage 1 and stage 2. Variant rs6598860 (RegulomeDB score of 2b) lies in the promoter of *ARID1A* and might affect the binding affinity of transcription machinery units on the promoter (RegulomeDB). Variant rs6598860 has also been associated with leptin level and birth length at nominal significance level (*P* <  = 0.05) in Europeans^20^. Variant rs4589135 has also been associated with High-Density Lipid (HDL) levels of European and mixed ancestry samples in Global Lipids Genetics Consortium (GLGC) study at nominal significance level (P <  = 0.05)^21^. It has also been associated with triglycerides levels in European population^22^.

Although no genetic study has linked *ARID1A* with adult or childhood obesity, a functional study involving *ARID1A* deficient mice showed higher expression of Interleukin 6 (IL6), a known inflammatory cytokine^23^. *ARID1A* is a transcription factor and its depletion has been reported to affect cholesterol synthesis as well as glycogen metabolism related proteins levels in ovarian cancer cell lines^24^. Study in skeletal muscle had shown positive correlation of expression of *ARID1A* with BMI in humans^25^. These evidences suggest that *ARID1A* may affect obesity through cytokines (IL6), adipokines (leptins) or lipids mediated lipid pathways.

We also found an association of rs3804562 in *KAT2B*, which codes for a histone acetyltransferase, with BMI. KAT2B knockdown mice showed a reduction in body weight and hyperglycemia in comparison to control mice^26^. Also, its role in gluconeogenesis and energy maintenance mechanism has been suggested by a previous study^27^.

In conclusion, our data revealed that common variants of *ARID1A* and *KAT2B* are associated with increased susceptibility to overweight/obesity in Indian urban adolescents. Our study had used overweight individuals along with obese individuals as case group, the effect size of identified associations can be underestimated and should be interpreted cautiously in case of obese subjects. Since, the current study aimed at investigating the important genes from chromatin modifiers pathway, and list of genes included in the study might not be an exhaustive list of all genes involved in the pathway. An exhaustive investigation of all the listed genes in literature might help to identify more genetic variants associated with childhood obesity in chromatin modifier genes. Although the case-control studies design limits the potential to identify the causal relationships, our study provides a lead for future investigations toward understanding the contribution of epigenetic modifiers in genetic predisposition to obesity in adolescents. This would help in understanding the molecular mechanisms and exploring therapeutic options toward prevention of childhood obesity.

## Methods

The study involved the participation of 3,530 adolescents (aged 11–17 years) including 2,539 normal-weight (NW group) and 991 overweight/obese (OW/OB group) participants. All the participants belonged to Indo-European ethnicity and were recruited from school health surveys in five different zones of Delhi (north, south, east, west, and central regions) and National Capital Region as described previously^19,28,29^. Prior permission from school authorities, informed consent from parents/guardians and verbal consent from participants themselves were obtained before participation in the study. The study plan was discussed in detail with school authorities for administrative approval. A written plan was circulated to the parents through the school. The study protocol was approved by ethics committees of CSIR-Institute of Genomics and Integrative Biology and All India Institute of Medical Sciences. The study was conducted according to principles of the Declarations of Helsinki. Anthropometric measurements including height, weight, waist circumference (WC) and hip circumference (HC) were taken using standard methods and BMI was calculated. Blood samples were drawn from participants after overnight fast and DNA was extracted as mentioned previously^30^. Participants were classified as normal weight and overweight/obese according to age- and sex-specific cutoffs provided by Cole *et al*., 2002^31^.

In stage 1, we initially selected 203 SNPs in 37 genes for genotyping after exhaustive literature survey on chromatin modifiers. Most of the genes included in this study were selected from You *et al*. who reviewed available literature till 2012 for epigenetic process-related genes in case of cancer^32^. We also included genes that were not included in this review but literature search revealed their involvement in the epigenetic process. The SNPs were selected on basis of their presence in functionally important regions of genes, previous reports of association with metabolic disorders and a minor allele frequency greater than or equal to 0.05. Genotyping was done on 1283 participants using Illumina Golden Gate assay (Illumina, San Diego, CA). Genotyping data were subjected to extensive quality control (QC). SNPs with genotype confidence score (confidence value assigned to each called genotype that ranges between 0 and 1 with less reliable call assigned lower value) less than 0.25 were removed. We also removed SNPs with GenTrain score (a statistical score that mimics evaluations made by a human expert’s visual and cognitive systems about clustering behavior of a locus, less reliable cluster assigned lower value) less than 0.6. SNPs with cluster separation score (cluster separation measurement between different genotypes for an SNP that ranges between 0 and 1) less than 0.4 and call rate < 0.9 were also removed. Further, SNPs with Hardy-Weinberg equilibrium *P* value less than 0.01 in any of the NW, OW/OB and combined sample groups were removed. Out of selected 203 SNPs in 35 genes, 5 SNPs (rs66797130, rs9909489, rs16834954, rs6576 and rs341530259) in 4 genes were non-polymorphic in our samples. In total 24 SNPs in 17 different genes failed in the assay. The final analysis was done on 1283 adolescent (830 NW and 453 OW/OB adolescent) for 179 SNPs from 35 genes, encoding DNA methylation enzymes, histone modifiers and chromatin modifiers (Supplementary Table 1). After QC, SNPs (n = 179) had a call rate of 98% and a concordance rate of 99.97% with 5% duplicate samples. Genotype frequencies for all the SNPs are provided in Supplementary Table 1.

Genotyping of 18 obesity-associated SNPs (17 direct SNPs and one proxy SNP, rs56315139 for rs6504550 as shown in Supplementary Table 2) from stage 1 was performed in 2247 adolescent (1709 normal weight and 538 overweight/obese) using iPLEX (Sequenom, San Diego, CA now Agena Bioscience, Hamburg, Germany). We failed to design primers for remaining SNPs using Assay Design Suite Agena (https://seqpws1.agenacx.com/AssayDesignerSuite.html) in a single plex. Stringent QC for the genotyped data was performed. We removed two SNPs with a call rate less than 90% and 3 SNPs with HWE *P*-value < 2.78 × 10^−3^ (0.05/18) during analysis in stage 2. Finally, we analyzed 13 SNPs in stage 2. The average genotyping success rate for remaining SNPs was 96% (range = 91–100%) with 99.7% consistency in genotyping with 10% duplicates.

Statistical analysis was performed using PLINK version 1.07 (http://pngu.mgh.harvard.edu/;purcell/plink)^33,34^ and R version 3.1.0. Genotype frequencies were checked for Hardy-Weinberg equilibrium using the χ2 test. Prior to analysis, internal age- and sex-specific z scores were calculated for continuous variables as described previously^31^. The z scores were inverse normal transformed to achieve normal distribution. Logistic regression analysis under a log-additive model adjusting for age and sex was performed to test the association of QC-passed SNPs with overweight/obesity in PLINK. Associations for continuous traits related to obesity were performed using linear regression model adjusted for age and sex assuming the additive mode of inheritance. Meta-analysis of summary statistics from stage 1 and stage 2 associations was performed using fixed-effect inverse variance method using METAL (http://www.sph.umich.edu/csg/abecasis/Metal/). A *P*-value of 6.33 × 10^–4^ after meta-analysis was considered significant after correcting for 79 independent loci (r^2^ < 0.8) for obesity and BMI. We did not correct for multiple phenotypes as all the tested phenotypes (obesity and BMI) are correlated with each other.

We have collected samples from a small geographical region that forms a homogenous cluster as shown by Dwivedi OP *et al*., 2012^30^. Principal component analysis of genetic data (Axiom™ Genome-Wide EUR 1 Array) available for 1095 participants revealed that our samples are genetically homogeneous (Supplementary Figure 2). The statistical power of the study was calculated using the log-additive model of inheritance of considering 24% prevalence of overweight/obesity^2^ for SNPs with allele frequency ranging between 0.05–0.50 and an effect size of 1.05–2 at α = 6.33 × 10^−4^.

Raw genotype data used in the study has been included in the manuscript as supplementary dataset S1.

## Electronic supplementary material

Supplementary Information

Dataset 1
