# Supplementary material for: Common variants of ARID1A and KAT2B are associated with obesity in Indian adolescents
Source: Sci Rep. 2018 Mar 2;8:3964. doi: 10.1038/s41598-018-22231-x (PMC5834613; doi:10.1038/s41598-018-22231-x)
Supplement: Supplementary file 1 — Supplementary Information [file 41598_2018_22231_MOESM1_ESM.pdf]

Common variants of *ARID1A* and *KAT2B* are associated with obesity in Indian adolescents

Anil K Giri[1, 2+], Vaisak Parekatt[1+], Om Prakash Dwivedi[1], Priyanka Banerjee[1], Khushdeep Bandesh[1, 2], Gauri Prasad[1, 2], Nikhil Tandon[3*], Dwaipayan Bharadwaj[1,2,# 4*]

Supplementary Table 1: List of selected SNPs and their association with overweight/obesity and ZBMI in urban Indian children in stage 1 of the study

												**Overweight/obesity**		**ZBMI**		
**S. No**	**Gene**	**SNP**	**Chr Position**	**Position in gene**	**Base Change**	**RA**	**AA Change**	**P HWE OW/OB**	**P HWE NW**	**RAF OW/OB**	**RAF NW**	**OR (95%CI)**	**P**	**β**	**SE**	**P**
1	*ARID1A*	rs6598860	27021684	5' near gene	T/C	T		0.82	0.56	0.31	0.76	1.37 (1.14-1.65)	**7.83x10^{-4}**	0.17	0.05	**2.12x10^{-4}**
2		rs11247594	27067417	intronic	A/G	G		0.77	0.21	0.57	0.5	1.30 (1.10-1.54)	**1.66x10^{-3}**	-0.12	0.04	**3.67x10^{-3}**
3		rs4589135	27041714	intronic	A/G	A		1	0.64	0.43	0.66	1.41(1.18-1.68)	**1.19x10^{-4}**	0.15	0.04	**6.03x10^{-4}**
4	*BAZ1A*	rs1959152	35237117	intronic	C/A	C		0.25	0.49	0.2	0.82	1.15 (0.94-1.40)	0.19	0.09	0.05	0.07
5		rs2275145	35242828	synonymous	T/C	T		0.39	1	0.43	0.59	1.08 (0.91-1.28)	0.37	0.01	0.04	0.79
6		rs3783313	35342838	intronic	A/G	A		0.12	0.93	0.27	0.74	1.10 (0.90-1.33)	0.36	0.02	0.05	0.62
7		rs13379337	35224430	intronic	C/T	C		0.69	0.44	0.39	0.62	1.05 (0.88-1.24)	0.61	-0.02	0.04	0.61
8	*BPTF*	rs6504550	65894542	intronic	T/C	C		0.4	0.47	0.67	0.6	1.33 (1.11-1.59)	**1.51x10^{-3}**	-0.13	0.04	**1.83x10^{-3}**
9		rs8074078	65838743	intronic	A/T	T		1	0.48	0.78	0.73	1.33(1.10-1.61)	**3.75x10^{-3}**	-0.12	0.05	**0.01**
10		rs4318247	65882172	intronic	C/G	G		0.89	0.93	0.78	0.73	1.32 (1.08-1.59)	**7.6x10^{-3}**	-0.13	0.05	**4.10x10^{-3}**
11		rs3935969	65881959	intronic	A/G	G		0.08	0	0.79	0.75	1.27 (1.02-1.56)	**0.03**	-0.11	0.05	**0.04**
12		rs4790974	65951107	intronic	T/A	A		0.35	1	0.77	0.74	1.20 (1.00-1.47)	0.05	-0.08	0.05	0.11
13		rs8071463	65938655	intronic	G/T	T		0.13	0.22	0.78	0.75	1.20 (0.99-1.47)	0.06	-0.08	0.05	0.09
14	*CERS2*	rs4451553	150937329	3' near gene	A/G	A		0.78	0.66	0.49	0.54	1.12 (0.95-1.33)	0.18	0.05	0.04	0.23
15	*CHD4*	rs2284325	6704213	intronic	T/A	A		0.79	0.92	0.78	0.76	1.06 (0.88-1.30)	0.52	-0.07	0.05	0.15
16	*CHD5*	rs3827728	6162407	3'-UTR	C/T	T		0.85	0.68	0.56	0.52	1.19 (1.01-1.41)	**0.04**	-0.08	0.04	0.06
17		rs747392	6222075	intronic	G/A	G		0.37	0.14	0.37	0.66	1.15 (0.97-1.37)	0.11	0.03	0.04	0.43
18		rs2843493	6184092	missense	A/G	A	S1539P	0.8	0.76	0.25	0.78	1.19 (0.98-1.45)	0.08	0.08	0.05	0.11
19		rs12565328	6209363	synonymous	G/T	G		0.85	0.2	0.48	0.54	1.12 (0.94-1.33)	0.19	0.06	0.04	0.17
20		rs9434662	6190215	intronic	G/A	A		0.08	1	0.72	0.71	1.08(0.91-1.43)	0.44	-0.09	0.04	**0.04**
21		rs2250358	6196869	synonymous	T/C	T		0.33	0.56	0.42	0.6	1.09 (0.92-1.29)	0.3	0.01	0.04	0.83
22		rs1883606	6207217	intronic	T/C	C		0.39	0.42	0.5	0.5	1.00 (0.84-1.18)	0.97	0	0.04	0.92
23	*CHD9*	rs16945073	53360273	3'-UTR	C/T	T		0.11	0.56	0.8	0.76	1.23 (1.01-1.52)	**0.04**	-0.09	0.05	0.08
24		rs6499548	53341748	missense	T/C	T	D2313E	0.9	0.3	0.25	0.79	1.22 (1.00-1.49)	0.05	0.05	0.05	0.35
25		rs7201808	53188669	intronic	C/T	C		0.84	0.48	0.4	0.63	1.18 (0.99-1.40)	0.07	0.04	0.04	0.33
26		rs12933421	53302383	intronic	A/G	A		0.5	0.14	0.44	0.6	1.17 (0.99-1.38)	0.06	0.05	0.04	0.25
27		rs1421069	53197934	intronic	G/T	G		0.39	0.35	0.44	0.6	1.17 (1.0-1.38)	0.06	0.04	0.04	0.27
28		rs2110843	53269316	intronic	T/G	T		0.25	0.22	0.43	0.6	1.16 (0.99-1.34)	0.07	0.04	0.04	0.31
29		rs11860987	53260888	intronic	C/A	C		0.38	0.14	0.43	0.6	1.16 (0.98-1.37)	0.08	0.04	0.04	0.29
30		rs8058720	53314679	intronic	C/A	C		0.78	0.1	0.48	0.55	1.17 (0.99-1.37)	0.07	0.03	0.04	0.48
31		rs8052283	53283966	synonymous	A/G	G		0.46	0.08	0.65	0.64	1.10 (0.92-1.32)	0.32	-0.02	0.04	0.63
32		rs3743771	53358439	missense	C/T	T	T2761A	0.25	0.43	0.71	0.72	1.00 (0.83-1.2)	0.98	-0.01	0.05	0.82
33		rs1420306	53297395	intronic	C/G	C		0.02	0.11	0.13	0.87	1.03 (0.81-1.30)	0.82	-0.02	0.06	0.73
34	*CHRAC1*	rs4246131	141526038	3'-UTR	A/G	G		0.33	0.43	0.78	0.77	1.04 (0.85-1.27)	0.68	-0.06	0.05	0.24
35	*DNMT1*	rs8101626	10246029	intronic	A/G	G		0.6	0.76	0.67	0.64	1.12 (0.94-1.35)	0.18	-0.01	0.04	0.77
36		rs2228611	10267077	synonymous	C/T	C		0.7	0.67	0.44	0.58	1.08 (0.92-1.28)	0.35	0.03	0.04	0.54

37		rs9305012	10260869	intronic	A/G	A		0.58	0.44	0.22	0.8	1.14 (0.93-1.40)	0.2	0.04	0.05	0.39
38		rs8111085	10162696	missense	A/G	A	Ile327Val	0.56	0.82	0.2	0.81	1.14 (0.93-1.40)	0.21	0.02	0.05	0.63
39		rs2241531	10271034	intronic	C/T	C		0.32	0.82	0.21	0.81	1.13 (0.92-1.39)	0.23	0.03	0.05	0.57
40	*DNMT3A*	rs6733868	25499867	intronic	C/T	C		0.77	0.5	0.44	0.63	1.31 (1.11-1.55)	**1.46x10^{-3}**	0.09	0.04	**0.03**
41		rs6739187	25513652	intronic	G/C	G		0.12	0.93	0.35	0.69	1.14 (0.96-1.35)	0.14	0.04	0.04	0.36
42		rs6722613	25539357	intronic	A/C	A		0.11	1	0.49	0.55	1.12 (0.95-1.31)	0.18	0.01	0.04	0.81
43		rs734693	25463871	intronic	G/T	T		0.63	0.89	0.56	0.55	1.05 (0.88-1.23)	0.57	0.01	0.04	0.84
44		rs2276598	25469502	synonymous	C/T	C		0.1	0.27	0.11	0.9	1.11 (0.85-1.44)	0.44	0.1	0.07	0.15
45		rs749131	25529624	intronic	T/C	T		0.43	0.77	0.4	0.61	1.04 (0.88-1.23)	0.64	-0.01	0.04	0.83
46		rs7594432	25482883	intronic	C/G	G		0.29	0.17	0.66	0.66	1.02 (0.85-1.20)	0.83	-0.02	0.04	0.64
47		rs10084238	25458379	intronic	T/C	T		1	0.72	0.46	0.54	1.01 (0.85-1.19)	0.95	-0.06	0.04	0.12
48	*DNMT3B*	rs6087990	31349908	5' near gene	G/A	G		1	0.33	0.35	0.68	1.14 (0.95-1.35)	0.15	0.03	0.04	0.5
49		rs6058891	31386347	synonymous	T/C	T		0.75	0.25	0.34	0.69	1.15 (0.97-1.37)	0.11	0.04	0.04	0.33
50		rs6058883	31365719	intronic	A/G	A		0.75	0.05	0.33	0.7	1.13 (0.95-1.34)	0.17	0.04	0.04	0.39
51		rs6088008	31382860	intronic	A/C	A		0.77	0.21	0.41	0.61	1.09 (0.93-1.29)	0.3	0.02	0.04	0.58
52		rs910084	31379665	intronic	C/T	C		0.92	0.46	0.41	0.61	1.06 (0.90-1.26)	0.48	0.02	0.04	0.66
53		rs2424932	31396536	3'-UTR	T/G	T		0.38	0.84	0.25	0.77	1.09 (0.90-1.31)	0.4	0.02	0.05	0.74
54		rs4911108	31375311	intronic	G/A	G		0.63	0.16	0.4	0.62	1.09 (0.93-1.29)	0.29	0.02	0.04	0.59
55		rs17123658	31390840	intronic	C/T	C		0.7	0.66	0.41	0.6	1.03 (0.87-1.22)	0.73	-0.01	0.04	0.74
56		rs910085	31383353	intronic	A/G	G		0.1	0.82	0.38	0.63	1.05 (0.88-1.26)	0.58	0.01	0.04	0.8
57	*EZH1*	rs7359598	40897470	5' near gene	T/C	T		0.24	0.1	0.42	0.61	1.12 (0.95-1.32)	0.2	0.05	0.04	0.22
58		rs2089115	40858841	intronic	C/G	G		0.51	0.49	0.54	0.51	1.14 (0.96-1.33)	0.12	-0.05	0.04	0.27
59		rs7215553	40896077	intronic	C/A	C		0.56	0.56	0.42	0.62	1.13 (0.96-1.34)	0.16	0.06	0.04	0.15
60		rs2242461	40861424	intronic	G/T	G		0.17	0.82	0.4	0.63	1.11 (0.93-1.31)	0.24	0.04	0.04	0.31
61		rs4792953	40873303	intronic	T/C	T		0.37	0.6	0.38	0.64	1.09 (0.92-1.29)	0.34	0.05	0.04	0.22
62		rs7214055	40853171	3'-UTR	T/A	T		0.34	0.85	0.11	0.89	1.05 (0.80-1.38)	0.72	-0.02	0.07	0.78
63	*EZH2*	rs757624	148503377	intronic	T/C	C		0.18	0.91	0.83	0.82	1.09 (0.88-1.35)	0.45	0.03	0.05	0.6
64		rs2177567	148578753	intronic	T/C	T		0.7	0.66	0.43	0.61	1.18 (1.00-1.39)	**0.04**	0.09	0.04	**0.02**
65		rs6464926	148519011	intronic	A/G	A		0.63	0.83	0.44	0.6	1.17 (0.99-1.38)	0.07	0.07	0.04	0.1
66		rs17171118	148517304	intronic	T/C	C		0.31	1	0.83	0.81	1.12 (0.90-1.39)	0.32	0.03	0.05	0.62
67		rs734004	148505460	intronic	T/G	G		0.11	0.17	0.62	0.6	1.10 (0.93-1.30)	0.26	-0.09	0.04	**0.03**
68		rs2072407	148508833	intronic	T/G	G		0.06	0.15	0.63	0.6	1.10 (0.93-1.30)	0.25	-0.08	0.04	**0.04**
69		rs9691534	148544919	intronic	G/A	A		0.03	0.75	0.69	0.68	1.05 (0.88-1.25)	0.57	-0.07	0.04	0.12
70		rs10274535	148543525	intronic	T/C	C		0.09	0.76	0.66	0.66	1.02 (0.85-1.20)	0.85	-0.06	0.04	0.16
71		rs2302427	148525904	missense	A/C	C	D193H	0.51	1	0.92	0.93	1.04 (0.76-1.42)	0.81	0.01	0.08	0.9
72	*HDAC1*	rs1741981	32756439	upstream	C/T	C		0.22	0.32	0.31	0.74	1.20 (1.01-1.44)	**0.04**	0.07	0.04	0.11
73	*HDAC2*	rs6568819	114270928	intronic	A/G	A		0.62	0.41	0.25	0.79	1.23 (1.01-1.49)	**0.04**	0.1	0.05	**0.04**
74		rs3757016	114260720	3'-UTR	T/C	C		0.3	0.21	0.65	0.61	1.16 (0.98-1.37)	0.08	-0.04	0.04	0.33
75		rs9481408	114265287	intronic	T/C	T		0.92	0.03	0.37	0.66	1.12 (0.95-1.33)	0.18	0.07	0.04	0.11
76		rs13213007	114282481	intronic	A/G	A		0.91	0.6	0.28	0.73	1.02 (0.85-1.23)	0.8	-0.04	0.05	0.39
77	*HDAC4*	rs1979449	239997710	intronic	C/T	C		1	0	0.38	0.65	1.16 (0.98-1.37)	0.08	0.05	0.04	0.22
78		rs3791500	240075545	intronic	A/G	A		0.36	0.76	0.24	0.78	1.12 (0.92-1.36)	0.24	0.01	0.05	0.81
79		rs3791406	240030484	intronic	G/A	G		0.68	1	0.22	0.8	1.11 (0.91-1.35)	0.32	0.02	0.05	0.65
80		rs4852010	239972883	3'-UTR	A/C	A		0.7	0.44	0.24	0.77	1.07 (0.88-1.29)	0.53	0.01	0.05	0.77
81		rs2898824	240243682	intronic	G/A	G		0.85	0.53	0.48	0.53	1.05 (0.89-1.24)	0.55	0.06	0.04	0.15
82		rs35291459	239134270	synonymous	T/C	C		0.07	1	0.99	0.99	1.02(0.51-2.04)	0.95	0.03	0.17	0.84
83		rs1063639	240003870	synonymous	T/G	G		0.22	0	0.63	0.62	1.02 (0.86-1.20)	0.82	0.04	0.04	0.36
84		rs678228	240127024	intronic	A/T	T		0.37	0.17	0.76	0.76	1.00 (0.83-1.21)	0.99	-0.04	0.05	0.37
85	*IKZF3*	rs10445308	37938047	intronic	T/C	T		0.25	0.94	0.43	0.63	1.27 (1.07-1.5)	**6.4x10^{-3}**	0.19	0.04	**9.29x10^{-6}**

86		rs9303277	37976469	intronic	A/C	A		0.22	0.83	0.45	0.6	1.23 (1.04-1.46)	**0.01**	0.18	0.04	**1.50x10^{-5}**
87		rs907092	37922259	synonymous	G/A	G		0.28	0.49	0.42	0.64	1.29 (1.09-1.53)	**3.56x10^{-3}**	0.2	0.04	**3.59x10^{-6}**
88		rs907091	37921742	3'-UTR	G/A	G		0.29	0.3	0.44	0.6	1.21 (1.02-1.43)	**0.03**	0.17	0.04	**3.93x10^{-5}**
89		rs7173954	41275014	intronic	G/A	G		1	1	0.28	0.75	1.2 (1.0-1.45)	0.06	0.1	0.05	**0.04**
90		rs12910027	41283817	intronic	G/A	G		0.22	0.26	0.49	0.53	1.07 (0.90-1.26)	0.43	0.04	0.04	0.38
91	*INO80*	rs12101934	41353079	intronic	C/A	C		0.13	0.23	0.49	0.53	1.05 (0.89-1.24)	0.58	0.04	0.04	0.35
92		rs8042088	41337487	intronic	A/G	G		0.22	0.94	0.53	0.52	1.01 (0.86-1.19)	0.86	-0.01	0.04	0.76
93		rs2306083	41347658	intronic	C/T	C		0.11	0.72	0.47	0.52	0.99 (0.84-1.17)	0.91	-0.01	0.04	0.74
94		rs12639078	20079878	5' near gene	T/A	T		0.9	0.3	0.26	0.75	1.07 (0.89-1.29)	0.47	0.09	0.05	**0.05**
95		rs2929401	20096735	intronic	C/T	C		0.4	0.83	0.47	0.58	1.21 (1.03-1.43)	**0.02**	0.1	0.04	**0.01**
96	*KAT2B*	rs3804562	20181028	intronic	T/C	C		0.63	0.11	0.57	0.53	1.18 (1.00-1.39)	**0.04**	-0.11	0.04	**7.37x10^{-3}**
97		rs11128935	20125393	intronic	T/C	T		0.85	0.19	0.43	0.62	1.18 (1.00-1.40)	**0.04**	0.1	0.04	**0.02**
98		rs3021408	20113830	synonymous	A/G	A		0.11	0.12	0.48	0.52	1.15 (0.98-1.36)	**0.04**	0.06	0.04	**0.17**
99		rs7617840	20162216	intronic	G/A	G		0.92	0.64	0.35	0.66	1.08 (0.91-1.29)	0.39	0.02	0.04	0.67
100	*KAT5*	rs535111	65485337	3'-UTR	A/G	A		0.72	0.36	0.28	0.75	1.14 (0.95-1.37)	0.16	-0.02	0.05	0.61
101		rs1151500	65481166	intronic	T/C	C		0.15	0.13	0.73	0.73	1.05 (0.88-1.27)	0.55	-0.05	0.04	0.29
102		rs10953468	104741842	intronic	G/T	G		0.6	0.39	0.24	0.8	1.25 (1.02-1.53)	**0.03**	0.06	0.05	0.25
103		rs2240455	104717517	synonymous	G/A	A		0.87	0	0.82	0.78	1.28 (1.03-1.59)	**0.03**	-0.08	0.05	0.12
104		rs2299299	104751419	intronic	A/G	G		0	0.62	0.8	0.77	1.20 (0.99-1.59)	0.07	-0.07	0.05	0.12
105	*MLL5*	rs12671368	104704837	intronic	G/A	A		0	0.21	0.79	0.76	1.14 (0.94-1.39)	0.17	-0.06	0.05	0.24
106		rs740297	104669891	intronic	C/T	T		0.01	0.7	0.79	0.76	1.16 (0.96-1.41)	0.12	-0.06	0.05	0.23
107		rs2097942	104725105	intronic	C/T	C		1	0.78	0.46	0.56	1.05 (0.89-1.24)	0.56	0	0.04	0.98
108		rs10260346	104697512	intronic	T/G	G		0	0.05	0.79	0.78	1.08 (0.88-1.30)	0.48	-0.03	0.05	0.56
109		rs1805736	9084931	intronic	T/A	A		0.5	0.02	0.57	0.56	1.06 (0.90-1.25)	0.49	-0.02	0.04	0.68
110		rs3809218	9066613	5' near gene	G/A	G		0.13	1	0.25	0.76	1.08 (0.89-1.30)	0.45	-0.01	0.05	0.89
111	*PHC1*	rs1805732	9090892	intronic	T/G	G		0.44	0.11	0.57	0.55	1.05 (0.89-1.23)	0.52	-0.02	0.04	0.71
112		rs1805734	9089020	intronic	G/A	G		0.08	0.48	0.28	0.73	1.06 (0.89-1.27)	0.51	0.02	0.04	0.66
113		rs1049925	9086898	missense	A/T	T		0.84	0.66	0.59	0.58	1.03 (0.87-1.22)	0.7	0	0.04	0.91
114		rs674636	33142871	intronic	G/A	A		1	1	0.95	0.94	1.09 (0.75-1.59)	0.66	-0.07	0.09	0.43
115	*RBBP4*	rs2762905	33137928	intronic	C/T	T		0.5	0.35	0.93	0.92	1.09 (0.79-1.49)	0.61	-0.06	0.08	0.48
116		rs1748046	33124120	intronic	G/C	C		0.72	0.79	0.93	0.93	1.06 (0.77-1.47)	0.71	-0.02	0.08	0.79
117		rs6950894	104652671	5' near gene	T/C	T		0.85	0.07	0.46	0.55	1.03 (0.88-1.22)	0.7	-0.01	0.04	0.74
118	*RP11-*	rs2925345	41311799	intronic	G/A	G		0.15	0.05	0.48	0.54	1.07 (0.91-1.27)	0.41	0.04	0.04	0.4
119	*325F22.3*	rs7977273	56583644	5' near gene	A/G	A		1	0.32	0.07	0.94	1.31 (0.95-1.82)	0.1	0.07	0.08	0.38
120		rs7960225	56564811	intronic	G/A	G		0.28	0.5	0.27	0.76	1.18 (0.97-1.43)	0.1	0.06	0.05	0.22
121		rs7316626	6691452	intronic	T/C	C		0.63	0.2	0.82	0.81	1.05 (0.85-1.30)	0.63	-0.08	0.05	0.11
122		rs7950873	77412851	missense	G/A	G	S475P	0.05	0.15	0.19	0.84	1.25 (1.01-1.55)	**0.04**	0.03	0.05	0.54
123		rs11820337	77531890	5' near gene	T/C	T		0.43	0.16	0.19	0.83	1.16 (0.93-1.44)	0.19	-0.02	0.06	0.68
124	*RSF1*	rs6592742	77376247	3'-UTR	C/T	T		0.19	1	0.88	0.87	1.08 (0.85-1.39)	0.54	0.04	0.06	0.47
125		rs4945203	77409568	synonymous	A/G	G		0.2	1	0.88	0.87	1.05 (0.83-1.35)	0.67	0.05	0.06	0.42
126		rs17135807	77395252	intronic	T/C	C		0.2	1	0.87	0.87	1.05 (0.83-1.33)	0.69	0.05	0.06	0.41
127		rs12420401	77471901	intronic	C/T	T		0.18	1	0.88	0.88	1.00 (0.78-1.28)	1	0.06	0.06	0.32
128		rs2592984	140470212	intronic	A/G	G		0.66	0.49	0.68	0.65	1.18 (0.99-1.41)	0.06	-0.05	0.04	0.21
129		rs2725787	140451236	intronic	G/A	A		0.57	0.62	0.54	0.51	1.16 (0.98-1.37)	0.08	-0.06	0.04	0.17
130	*SETD7*	rs7680948	140447105	intronic	G/A	A		0.71	0.47	0.74	0.72	1.18 (0.97-1.43)	0.1	-0.07	0.05	0.13
131		rs17050790	239134270	intronic	G/A	A		1	0.44	0.99	0.97	2.00 (1.04-3.70)	**0.04**	-0.25	0.14	0.08
132		rs1026049	140430208	3'-UTR	G/T	T		0.36	0.74	0.8	0.8	1.04 (0.85-1.28)	0.72	-0.03	0.05	0.52
133		rs2725772	140438033	intronic	A/G	G		0.29	0.32	0.8	0.8	1.02 (0.83-1.25)	0.86	-0.03	0.05	0.57
134	*SETDB1*	rs2271076	150916657	intronic	T/C	T		0.92	0.94	0.48	0.54	1.11 (0.94-1.31)	0.23	0.05	0.04	0.26

135		rs4970986	150907955	intronic	T/C	T		0.71	1	0.49	0.53	1.10 (0.93-1.30)	0.26	0.04	0.04	0.38
136	*SETDB2*	rs1543513	50034684	intronic	G/A	A		0.75	0.08	0.66	0.63	0.86 (0.72-1.03)	0.1	-0.02	0.04	0.73
137		rs11619265	50057633	synonymous	A/G	G		0.84	0.33	0.66	0.63	1.12 (0.94-1.33)	0.2	0	0.04	0.92
138		rs9316454	50042484	intronic	G/A	A		0.83	0.64	0.69	0.67	1.09 (0.91-1.30)	0.34	0.01	0.04	0.84
139		rs2057413	50057097	missense	T/C	T	V183M	0.24	0.58	0.28	0.74	1.10 (0.91-1.32)	0.33	0.04	0.05	0.42
140		rs7998427	50050620	missense	C/A	C	E117G	0.7	0.61	0.42	0.6	1.04 (0.88-1.23)	0.63	0.01	0.04	0.85
141	*SMARCA2*	rs7040790	2013962	5' near gene	C/G	C		0.35	0.1	0.34	0.66	1.00 (0.84-1.19)	1	0.01	0.04	0.86
142		rs11789313	2193975	3' near gene	G/A	G		0.17	0.04	0.28	0.71	1.02 (0.85-1.20)	0.86	0.02	0.04	0.63
143		rs10811378	2071389	intronic	T/C	T		0.83	0.29	0.31	0.72	1.17 (0.97-1.40)	0.09	0.04	0.04	0.41
144		rs10964500	2034440	intronic	G/A	A		0.49	0.04	0.84	0.81	1.14 (0.92-1.41)	0.22	-0.04	0.05	0.44
145		rs2376306	2143489	intronic	G/C	G		0.22	0.52	0.45	0.57	1.10 (0.93-1.29)	0.29	0.02	0.04	0.63
146		rs4741638	2050844	intronic	C/T	T		0.87	0.12	0.82	0.81	1.10 (0.89-1.35)	0.37	-0.04	0.05	0.48
147		rs2296212	2191309	missense	C/T	C	D1528E	0.29	0.03	0.28	0.73	1.05 (0.88-1.25)	0.61	0.03	0.05	0.47
148		rs3829069	2056563	intronic	G/A	G		0.14	0.52	0.26	0.75	1.04 (0.86-1.25)	0.69	0.03	0.05	0.53
149		rs3793516	2174095	intronic	G/A	A		0.58	0.52	0.7	0.69	1.05 (0.88-1.25)	0.59	-0.02	0.04	0.68
150		rs6475500	2116037	synonymous	C/T	C		0.79	0.02	0.1	0.91	1.12 (0.84-1.49)	0.44	0.05	0.07	0.47
151		rs10964468	2029018	5'-UTR	T/C	C		1	0.19	0.93	0.93	1.01 (0.74-1.37)	0.96	0.02	0.08	0.79
152	*SMARCA5*	rs4690791	144448347	intronic	A/G	A		0.67	0.71	0.2	0.83	1.20 (0.97-1.47)	0.09	0.06	0.05	0.22
153		rs3924846	144458362	intronic	G/A	G		0.63	0	0.49	0.51	1.07 (0.91-1.26)	0.4	0.03	0.04	0.4
154		rs7698480	144453500	intronic	T/C	T		0.51	0.02	0.49	0.51	1.07 (0.91-1.26)	0.4	0.03	0.04	0.4
155		rs17017856	144463445	intronic	C/T	C		0.57	0	0.49	0.52	1.06 (0.90-1.25)	0.46	0.03	0.04	0.42
156		rs12512157	143521582	synonymous	T/C	T		0.64	0.03	0.49	0.52	1.07 (0.91-1.26)	0.41	0.04	0.04	0.37
157		rs7692376	144466050	intronic	T/C	T		0.85	0.01	0.49	0.51	1.06 (0.90-1.24)	0.51	0.02	0.04	0.63
158		rs1510882	144435937	intronic	G/A	A		0.82	0.19	0.7	0.69	1.06 (0.89-1.27)	0.51	-0.01	0.04	0.86
159	*SMARCB1*	rs2073387	24129005	5' near gene	G/C	G		0.54	0.49	0.19	0.82	1.00 (0.81-1.23)	0.99	0.01	0.05	0.89
160		rs17003998	24176053	intronic	T/C	C		0.11	0.32	0.77	0.74	1.19 (0.99-1.45)	**0.04**	-0.06	0.05	**0.2**
161		rs2070456	24158697	intronic	G/A	G		0.92	0.65	0.4	0.63	1.13 (0.95-1.35)	0.15	0.05	0.04	0.26
162		rs2186370	24171305	intronic	C/T	T		0.92	0.06	0.56	0.54	1.09 (0.92-1.28)	0.33	-0.02	0.04	0.62
163		rs2073389	24133493	intronic	A/G	A		0.92	0.02	0.37	0.63	1.02 (0.85-1.22)	0.82	0.01	0.04	0.89
164	*SMARCC1*	rs1014228	47652639	intronic	T/C	T		0.26	0.21	0.45	0.54	1.00 (0.85-1.18)	1	-0.03	0.04	0.48
165		rs11714840	47747099	intronic	A/G	A		0.69	0.31	0.38	0.62	1.01 (0.84-1.20)	0.94	0.01	0.04	0.85
166		rs6786001	47759990	intronic	C/G	C		0.15	0.15	0.43	0.56	1.01 (0.85-1.19)	0.95	-0.03	0.04	0.51
167		rs2293226	47626782	-	A/T	A		0.15	0.14	0.44	0.54	0.97 (0.82-1.15)	0.73	-0.04	0.04	0.36
168		rs35817317	47670712	synonymous	C/T	C		0.29	0.91	0.19	0.82	1.09 (0.88-1.35)	0.41	0.06	0.05	0.24
169		rs1486915	47822543	intronic	A/G	A		0.25	0.12	0.43	0.56	1.02 (0.87-1.20)	0.77	-0.03	0.04	0.47
170	*SMARCE1*	rs757412	38784927	3'-UTR	G/A	A		0.38	0.79	0.88	0.85	1.35 (1.05-1.72)	**0.02**	-0.19	0.06	**$1.17x10^{-3}$**
171		rs16966018	38788153	intronic	A/G	G		0.53	0.6	0.87	0.84	1.32(1.03-1.67)	**0.03**	-0.17	0.06	**$2.88x10^{-3}$**
172		rs1474454	38791481	intronic	G/A	G		0.92	0.72	0.45	0.57	1.1 (0.93-1.3)	0.26	0.05	0.04	0.21
173		rs3752026	38804561	5' near gene	G/A	G		0.92	0.35	0.43	0.59	1.06 (0.90-1.26)	0.47	0.04	0.04	0.3
174		rs6503545	38798073	intronic	T/C	T		0.92	0.78	0.44	0.57	1.09 (0.92-1.29)	0.31	0.05	0.04	0.21
175	*SUV39H2*	rs11594111	14945406	3'-UTR	A/G	A		0.04	0.16	0.09	0.92	1.16 (0.87-1.55)	0.31	0.04	0.07	0.59
176		rs17353856	14941654	synonymous	C/G	C		0.02	0.16	0.09	0.92	1.15 (0.86-1.54)	0.34	0.04	0.07	0.61
177		rs10430783	14941965	intronic	G/A	G		0	0.06	0.47	0.53	1.01 (0.85-1.19)	0.89	0.02	0.04	0.62
178	*TAF6L*	rs633742	62545346	intronic	A/T	T		0.88	0.02	0.8	0.77	1.22 (1.00-1.49)	0.05	-0.09	0.05	0.08
179		rs11231210	62543180	intronic	T/C	C		1	1	0.94	0.94	1.04(0.73-1.49)	0.8	0.03	0.09	0.75

AA Change: Amino Acid Change, HWE: Hardy–Weinberg equilibrium, RAF: Risk Allele Frequency, OR: Odds Ratio, SE: Standard Error

Supplementary Table 2: List of genotyped SNPs in stage 2 of study

S. No	SNP	Gene	Remarks
1	rs6598860	*ARID1A*	
2	rs4589135	*ARID1A*	
3	rs11247594	*ARID1A*	Call rate <90%
4	rs6733868	*DNMT3A*	Call rate <90%
5	rs3021408	*KAT2B*	
6	rs11128935	*KAT2B*	
7	rs3804562	*KAT2B*	
8	rs2929401	*KAT2B*	HWE failed
9	rs6568819	*HDAC2*	
10	rs2177567	*EZH2*	
11	rs907092	*IKZF3*	
12	rs9303277	*IKZF3*	HWE failed
13	rs8074078	*BPTF*	
14	rs3935969	*BPTF*	
15	rs6504550	*BPTF*	Proxy for rs56315139
16	rs4318247	*BPTF*	HWE failed
17	rs17003998	*SMARCE1*	
18	rs757412	*SMARCE1*	

Supplementary Table 3: The meta-analyzed results analyzed SNPs for adiposity measures

			Stage 1		Stage 2		META-ANALYSIS			
**Genes**	**Obesity SNPs**	**RA/OA**	**OR(95%CI)**	**P**	**OR(95%CI)**	**P**	**OR(95%CI)**	**P**	**Het P**	**Dir**
ARID1A	rs6598860	A/G	1.37(1.36-1.38)	$7.83x10^{-4}$	1.19 (1.01 - 1.4)	0.04	1.27 (1.14 - 1.39)	$1.58x10^{-4}$	0.26	++
ARID1A	rs4589135	G/A	1.41(1.40-1.42)	$1.19x10^{-4}$	1.11 (0.96 - 1.28)	0.15	1.22 (1.08 – 1.41)	$3.72x10^{-4}$	0.04	++
KAT2B	rs3804562	G/A	1.18(1.17-1.18)	0.04	1.14 (0.99 - 1.32)	0.08	1.15 (1.03 – 1.32)	$8.93x10^{-3}$	0.76	++
BPTF	rs6504550	A/G	1.26(1.26-1.27)	$1.51x10^{-3}$	1.05 (0.91 - 1.22)	0.48	1.16 (1.05 - 1.27)	0.01	0.05	++
HDAC2	rs6568819	T/C	1.23(1.22-1.23)	0.04	1.12 (0.95 - 1.32)	0.16	1.17 (1.04 - 1.29)	0.02	0.50	++
KAT2B	rs3021408	G/A	1.16(1.15-1.16)	0.04	1.10 (0.96 - 1.27)	0.16	1.12 (1.01 – 1.28)	0.03	0.67	++
SMARCE1	rs757412	C/T	1.35(1.33-1.35)	0.02	1.06 (0.86 - 1.32)	0.55	1.18 (0.99 - 1.45)	0.04	0.16	++
BPTF	rs8074078	A/G	1.33(1.33-1.35)	$3.75x10^{-3}$	0.96 (0.82 - 1.14)	0.67	1.1 (0.98 - 1.22)	0.13	0.01	+-
KAT2B	rs11128935	A/G	1.18(1.18-1.19)	0.04	1.02 (0.88 - 1.18)	0.77	1.09 (0.98 - 1.2)	0.13	0.20	++
IKZF3	rs907092	T/C	1.29(1.28-1.30)	$3.56x10^{-3}$	0.96 (0.83 - 1.12)	0.60	1.09 (0.98 - 1.2)	0.13	0.01	+-
SMARCE1	rs17003998	A/G	1.19(1.19-1.20)	0.04	0.82(0.70-0.95)	0.01	1.06 (0.94 - 1.18)	0.37	$2.72x10^{-4}$	+-
BPTF	rs3935969	A/G	1.26(1.26-1.27)	0.03	0.95(0.81-1.12)	0.57	1.06 (0.93 - 1.18)	0.41	0.04	+-
EZH2	rs2177567	A/G	1.18(1.17-1.18)	0.04	0.90(0.78-1.04)	0.15	1.01 (0.9 - 1.12)	0.86	0.02	+-
**Genes**	**BMI SNPs**	**RA/OA**	**β(SE)**	**P**	**β(SE)**	**P**	**β(SE)**	**P**	**Het P**	**Dir**
ARID1A	rs6598860	A/G	0.17(0.05)	$2.12x10^{-4}$	0.09(0.03)	0.01	0.12(0.03)	$1.16 x10^{-5}$	0.18	++
ARID1A	rs4589135	G/A	0.15(0.04)	$6.03x10^{-4}$	0.08(0.03)	0.01	0.1(0.02)	$3.57x10^{-5}$	0.20	++
KAT2B	rs3804562	G/A	0.11(0.04)	$7.37x10^{-3}$	0.10(0.03)	$6.20x10^{-4}$	0.11(0.02)	$1.35x10^{-4}$	0.86	++
BPTF	rs6504550	A/G	0.13(0.04)	$1.83x10^{-3}$	0(0.03)	0.90	0.05(0.02)	0.05	0.01	++

HDAC2	rs6568819	T/C	0.10(0.05)	0.04	0.04(0.04)	0.24	0.06(0.03)	0.03	0.33	++
KAT2B	rs3021408	G/A	0.06(0.04)	0.17	0.07(0.03)	0.01	0.07(0.02)	3.92×10^{-3}	0.70	++
SMARCE1	rs757412	C/T	0.19(0.06)	1.17×10^{-3}	0.06(0.04)	0.16	0.11(0.04)	2.12×10^{-3}	0.08	++
BPTF	rs8074078	A/G	0.12(0.05)	0.01	-0.01(0.03)	0.83	0.04(0.03)	0.20	0.03	+-
KAT2B	rs11128935	A/G	0.10(0.04)	0.02	0.08(0.03)	0.01	0.09(0.02)	3.21×10^{-4}	0.72	++
IKZF3	rs907092	T/C	0.20(0.04)	3.59×10^{-6}	0.03(0.03)	0.43	0.09(0.03)	6.83×10^{-4}	1.04×10^{-3}	++
SMARCE1	rs17003998	A/G	-0.06(0.05)	0.20	0.06(0.03)	0.08	0.02(0.03)	0.47	0.04	-+
BPTF	rs3935969	A/G	0.11(0.05)	0.04	-0.01(0.03)	0.86	0.03(0.03)	0.35	0.07	+-
EZH2	rs2177567	A/G	0.09(0.04)	0.02	-0.01(0.03)	0.79	0.03(0.02)	0.26	0.05	+-
**Genes**	**WEIGHT SNPs**	**RA/OA**	**β(SE)**	**P**	**β(SE)**	**P**	**β(SE)**	**P**	**Het P**	**Dir**
ARID1A	rs6598860	A/G	0.18(0.05)	5.56×10^{-4}	0.11(0.03)	1.69×10^{-3}	0.13(0.03)	5.87×10^{-6}	0.25	++
ARID1A	rs4589135	G/A	0.18(0.05)	1.55×10^{-4}	0.09(0.03)	3.25×10^{-3}	0.12(0.03)	6.12×10^{-6}	0.10	++
KAT2B	rs3804562	G/A	0.11(0.05)	0.02	0.1(0.03)	1.29×10^{-3}	0.1(0.03)	6.40×10^{-5}	0.79	++
BPTF	rs6504550	A/G	0.10(0.05)	0.03	-0.02(0.03)	0.47	0.02(0.03)	0.56	0.03	+-
HDAC2	rs6568819	T/C	0.14(0.06)	0.01	0.06(0.03)	0.08	0.08(0.03)	5.35×10^{-3}	0.25	++
KAT2B	rs3021408	G/A	0.09(0.04)	0.03	0.08(0.03)	0.01	0.08(0.02)	8.13×10^{-4}	0.76	++
SMARCE1	rs757412	C/T	0.18(0.07)	7.83×10^{-3}	0.04(0.04)	0.40	-0.08(0.04)	0.03	0.08	++
BPTF	rs8074078	A/G	0.09(0.05)	0.07	0.01(0.03)	0.86	0.03(0.03)	0.27	0.16	++
KAT2B	rs11128935	A/G	0.12(0.05)	0.01	0.07(0.03)	0.02	0.09(0.03)	6.84×10^{-4}	0.45	++
IKZF3	rs907092	T/C	0.20(0.05)	3.64×10^{-5}	0.03(0.03)	0.33	0.08(0.03)	2.08×10^{-3}	0.00	++
SMARCE1	rs17003998	A/G	-0.10(0.05)	0.07	0.04(0.03)	0.21	0(0.03)	0.91	0.03	-+

BPTF	rs3935969	A/G	0.08(0.06)	0.17	0.01(0.03)	0.79	0.03(0.03)	0.36	0.28	++
EZH2	rs2177567	A/G	0.11(0.05)	0.02	0.02(0.03)	0.45	0.05(0.03)	0.06	0.12	++
**Genes**	**WC SNPs**		**β(SE)**	**P**	**(SE)**	**P**	**β(SE)**	**P**	**Het P**	**Dir**
ARID1A	rs6598860	A/G	0.11(0.03)	$9.20x10^{-4}$	0.12(0.04)	$1.19x10^{-3}$	0.11(0.02)	$3.43x10^{-6}$	0.90	++
ARID1A	rs4589135	G/A	0.10(0.03)	$1.32x10^{-3}$	0.11(0.03)	$8.35x10^{-4}$	0.1(0.02)	$3.52x10^{-6}$	0.84	++
KAT2B	rs3804562	G/A	0.11(0.03)	0.15	0.1(0.03)	$7.66x10^{-4}$	0.07(0.02)	$7.74x10^{-4}$	0.15	++
BPTF	rs6504550	A/G	0.07(0.03)	0.02	0.03(0.03)	0.39	0.05(0.02)	0.02	0.32	++
HDAC2	rs6568819	T/C	0.08(0.03)	0.02	0.04(0.04)	0.24	0.06(0.03)	0.01	0.44	++
KAT2B	rs3021408	G/A	0.05(0.03)	0.06	0.07(0.03)	0.02	0.06(0.02)	$3.18x10^{-3}$	0.69	++
SMARCE1	rs757412	C/T	0.15(0.04)	$3.77x10^{-4}$	0.06(0.05)	0.22	-0.11(0.03)	$5.51x10^{-4}$	0.13	++
BPTF	rs8074078	A/G	0.05(0.03)	0.14	-0.01(0.04)	0.80	0.02(0.02)	0.37	0.23	+-
KAT2B	rs11128935	A/G	0.07(0.03)	0.02	0.07(0.03)	0.02	0.07(0.02)	$7.56x10^{-4}$	0.91	++
IKZF3	rs907092	T/C	0.14(0.03)	$7.15x10^{-6}$	0.04(0.03)	0.17	0.09(0.02)	$2.41x10^{-5}$	0.04	++
SMARCE1	rs17003998	A/G	-0.05(0.03)	0.12	0.03(0.03)	0.45	-0.01(0.02)	0.58	0.10	-+
BPTF	rs3935969	A/G	0.04(0.04)	0.31	-0.01(0.03)	0.83	0.01(0.03)	0.59	0.38	+-
EZH2	rs2177567	A/G	0.07(0.03)	0.02	0.02(0.03)	0.53	0.05(0.02)	0.03	0.25	++
**Genes**	**HC SNPs**		**β(SE)**	**P**	**β(SE)**	**P**	**β(SE)**	**P**	**Het P**	**Dir**
ARID1A	rs6598860	A/G	0.07(0.03)	0.01	0.1(0.04)	0.01	0.08(0.02)	$2.38x10^{-4}$	0.51	++
ARID1A	rs4589135	G/A	0.07(0.03)	$8.86x10^{-3}$	0.07(0.03)	0.04	0.07(0.02)	$9.43x10^{-4}$	0.93	++
KAT2B	rs3804562	G/A	0.02(0.03)	0.47	-0.09(0.03)	$2.59x10^{-3}$	0.05(0.02)	0.01	0.06	++

BPTF	rs6504550	A/G	0.07(0.03)	0.01	0(0.03)	0.98	0.04(0.02)	0.05	0.12	++
HDAC2	rs6568819	T/C	0.07(0.03)	0.02	0.07(0.04)	0.05	0.07(0.02)	2.36×10^{-3}	1.00	++
KAT2B	rs3021408	G/A	0.04(0.02)	0.13	0.09(0.03)	4.15×10^{-3}	0.06(0.02)	2.82×10^{-3}	0.20	++
SMARCE1	rs757412	C/T	0.10(0.04)	7.28×10^{-3}	0.05(0.05)	0.27	0.08(0.03)	0.01	0.43	++
BPTF	rs8074078	A/G	0.06(0.03)	0.03	0.01(0.04)	0.79	0.04(0.02)	0.06	0.24	++
KAT2B	rs11128935	A/G	0.04(0.03)	0.09	0.08(0.03)	0.01	0.06(0.02)	4.45×10^{-3}	0.39	++
IKZF3	rs907092	T/C	0.11(0.03)	1.68×10^{-5}	0.05(0.03)	0.16	0.09(0.02)	2.20×10^{-5}	0.11	++
SMARCE1	rs17003998	A/G	0.07(0.03)	0.08	-0.02(0.03)	0.47	0.02(0.02)	0.40	0.09	+-
BPTF	rs3935969	A/G	0.05(0.03)	0.12	0.01(0.04)	0.86	0.03(0.02)	0.21	0.36	++
EZH2	rs2177567	A/G	0.05(0.03)	0.06	0.03(0.03)	0.32	0.04(0.02)	0.03	0.67	++

Change in z score per increase in risk allele has been represented as β. Associations that remained significant has been highlighted in bold text. SE is standard error and P represents association p value. The parameters have been calculated by linear regression analysis adjusting for age and sex. Het P represents the heterogeneity in effect size between stage 1 and stage 2. CI: Confidence Interval

Supplementary Figure 1: Statistical power of study

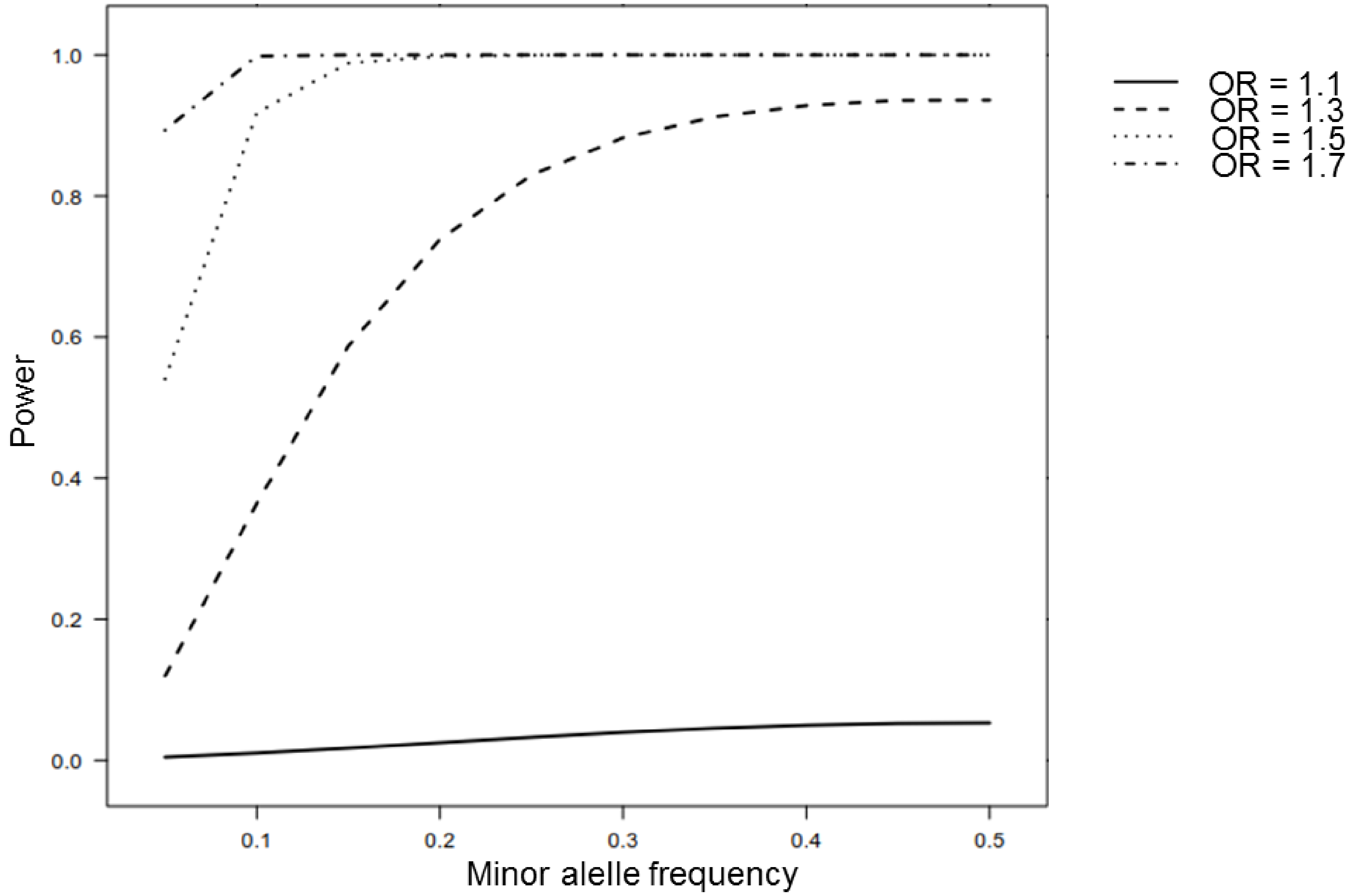

The power was calculated for allele frequencies ranging from 0.05 to 0.50 at different odds ratios from 1.10 to 1.40 assuming log-additive model of inheritance and 24% prevalence of disease at $\alpha=6.33\times10^{-4}$.

Supplementary Figure 2: Scatter plot showing the clustering of 1095 study participants on the basis of first two principal component of genetic data

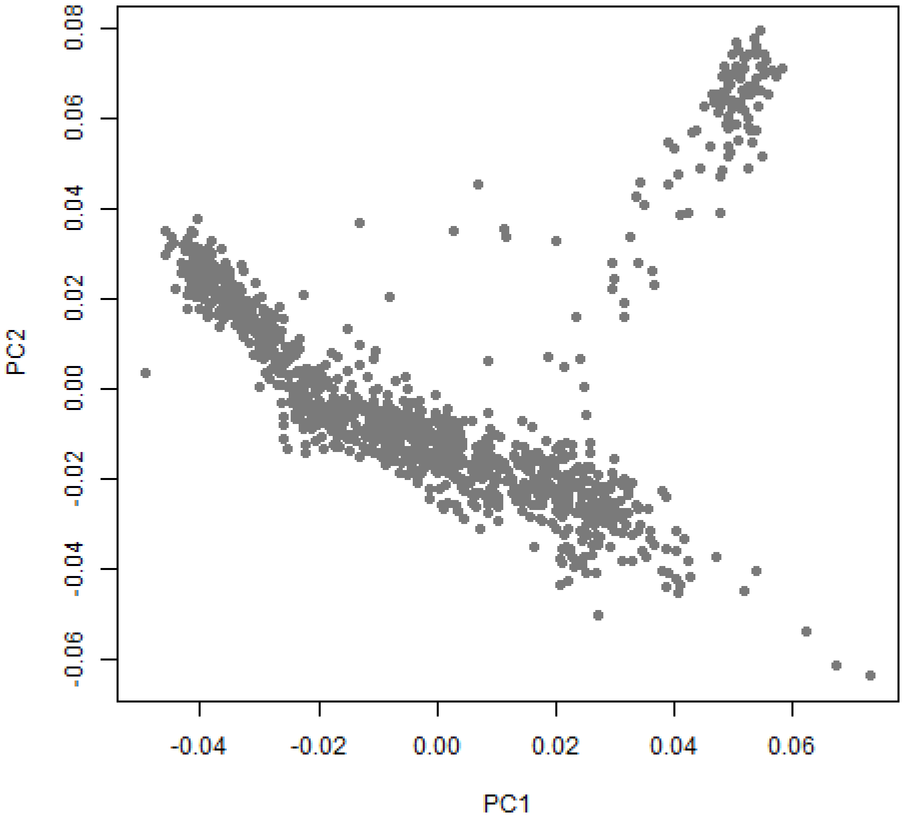

Whole genome genotyping data for Axiom™ Genome-Wide EUR 1 Array was available for 1095 study participants included in this study. Principal components were calculated using GCTA tool and plot was created using R. Plot represents that there is less genetic heterogeneity between study participants.
